# Disparities in United States hospitalizations for serious infections in patients with and without opioid use disorder: A nationwide observational study

**DOI:** 10.1371/journal.pmed.1003247

**Published:** 2020-08-07

**Authors:** June-Ho Kim, Danielle R. Fine, Lily Li, Simeon D. Kimmel, Long H. Ngo, Joji Suzuki, Christin N. Price, Matthew V. Ronan, Shoshana J. Herzig

**Affiliations:** 1 Division of General Internal Medicine and Primary Care, Department of Medicine, Brigham and Women’s Hospital, Boston, Massachusetts, United States of America; 2 Harvard Medical School, Boston, Massachusetts, United States of America; 3 Ariadne Labs, Brigham and Women’s Hospital and Harvard T.H. Chan School of Public Health, Boston, Massachusetts, United States of America; 4 Division of General Internal Medicine, Department of Medicine, Massachusetts General Hospital, Boston, Massachusetts, United States of America; 5 Division of Rheumatology, Immunology and Allergy, Department of Medicine, Brigham and Women’s Hospital, Boston, Massachusetts, United States of America; 6 Section of Infectious Diseases, Department of Medicine, Boston Medical Center, Boston, Massachusetts, United States of America; 7 Section of General Internal Medicine, Department of Medicine, Boston Medical Center, Boston, Massachusetts, United States of America; 8 Division of General Medicine, Department of Medicine, Beth Israel Deaconess Medical Center, Boston, Massachusetts, United States of America; 9 Department of Psychiatry, Brigham and Women’s Hospital, Boston, Massachusetts, United States of America; 10 Brigham and Women’s Physicians Organization, Brigham and Women’s Hospital, Boston, Massachusetts, United States of America; 11 Department of Medicine, West Roxbury VA Medical Center, Veterans Affairs Boston Healthcare System, Boston, Massachusetts, United States of America; Vanderbilt University Medical Center, UNITED STATES

## Abstract

**Background:**

Patients with opioid use disorder (OUD) who are hospitalized for serious infections requiring prolonged intravenous antibiotics may face barriers to discharge, which could prolong hospital length of stay (LOS) and increase financial burden. We investigated differences in LOS, discharge disposition, and charges between hospitalizations for serious infections in patients with and without OUD.

**Methods and findings:**

We utilized the 2016 National Inpatient Sample—a nationally representative database of all discharges from US acute care hospitals. The population of interest was all hospitalizations for infective endocarditis, epidural abscess, septic arthritis, or osteomyelitis. The exposure was OUD, and the primary outcome was LOS until discharge, assessed by using a competing risks analysis to estimate adjusted hazard ratios (aHRs). Adjusted odds ratio (aOR) of discharge disposition and adjusted differences in hospital charges were also reported. Of 95,470 estimated hospitalizations for serious infections (infective endocarditis, epidural abscess, septic arthritis, and osteomyelitis), the mean age was 49 years and 35% were female. 46% had Medicare (government-based insurance coverage for people age 65+ years), and 70% were non-Hispanic white. After adjustment for potential confounders, OUD was associated with a lower probability of discharge at any given LOS (aHR 0.61; 95% CI 0.59–0.63; *p <* 0.001). OUD was also associated with lower odds of discharge to home (aOR 0.38; 95% CI 0.33–0.43; *p <* 0.001) and higher odds of discharge to a post-acute care facility (aOR 1.85; 95% CI 1.57–2.17; *p <* 0.001) or patient-directed discharge (also referred to as “discharge against medical advice”) (aOR 3.47; 95% CI 2.80–4.29; *p <* 0.001). There was no significant difference in average total hospital charges, though daily hospital charges were significantly lower for patients with OUD. Limitations include the potential for unmeasured confounders and the use of billing codes to identify cohorts.

**Conclusions:**

Our findings suggest that among hospitalizations for some serious infections, those involving patients with OUD were associated with longer LOS, higher odds of discharge to post-acute care facilities or patient-directed discharge, and similar total hospital charges, despite lower daily charges. These findings highlight opportunities to improve care for patients with OUD hospitalized with serious infections, and to reduce the growing associated costs.

## Abstract 2

In a study of nationally representative hospital discharges in the US included in the 2016 National Inpatient Sample database, June-Ho Kim and colleagues identify associations between opioid use disorder and factors related to hospitalization for the treatment of serious bacterial infections.

## Author summary

**Why was this study done?:**

- There has been an increase in hospitalizations in the United States for serious infections among patients with opioid use disorder.
- These infections typically require several weeks of intravenous antibiotics, which can eventually be administered at home or at facilities outside of the hospital if the patient has no other inpatient needs. However, patients with opioid use disorder are often kept in the hospital to finish their treatment, which could have significant financial costs, use hospital resources, and cause patient harm.
- We conducted this study in order to understand differences in length of hospital stay, type of hospital discharge, and related financial charges between patients with and without opioid use disorder in the US.

**What did the researchers do and find?:**

- We analyzed a cross-sectional dataset representative of all hospitalizations for serious infections in the US during 2016 and compared length of hospital stay, type of hospital discharge, and financial charges.
- From 95,470 estimated hospitalizations, patients with opioid use disorder stayed an average of 4 days longer in the hospital compared to those without the disorder, with a 39% lower likelihood of discharge from the hospital at any given length of stay.
- Patients with opioid use disorder were less likely to be discharged home and more likely to be sent to a post-acute care facility or to self-discharge. Although daily hospital charges were lower for patients with opioid use, charges for the total hospitalization were similar given the longer stays.

**What do these findings mean?:**

- These disparities in hospital stays and destinations after discharge suggest that people with opioid use disorder may lack post-discharge options such as skilled nursing facilities or home care for antibiotic infusions and thus remain in the hospital longer than their counterparts without opioid use disorder.
- Consideration should be given to expanding discharge options for people with opioid use disorder to reduce costly hospital stays and provide equitable care for serious infections.

## Introduction

One of the many downstream consequences of the opioid crisis has been a marked increase in the incidence and associated costs of hospitalizations for serious bacterial infections associated with injection drug use such as endocarditis, osteomyelitis, septic arthritis, and epidural abscesses [1–6]. Treatment of these infections usually requires a prolonged course of intravenous (IV) antibiotics, which can often be completed from home in patients without another indication for a rehabilitation stay [7–11]. However, because this treatment involves sustained IV access, clinicians may be reluctant to discharge patients with opioid use disorder (OUD) to home, and home infusion companies may be reluctant to provide home services [12,13]. In addition, people with OUD face barriers to accessing post-acute care (PAC) facilities [13]. Taken together, these factors may result in longer hospital length of stay (LOS) and increased utilization of PAC facilities among people with OUD-associated infections, with important financial implications for hospitals and payers [14,15].

Prior research and clinical experience suggest that patients with OUD who are hospitalized with endocarditis or undergoing surgery for complications related to endocarditis have longer LOS and more patient-directed discharges (also referred to as “discharges against medical advice”) compared to those without OUD [2,16–19]. To our knowledge, there is no research to date assessing national differences in healthcare utilization of patients with and without OUD who are hospitalized for serious infections requiring prolonged IV access.

Using nationally representative data, we compared markers of healthcare utilization in hospitalizations of patients with a serious infection with and without OUD. We hypothesized that, even when accounting for differences in baseline characteristics, hospitalizations for serious infection among patients with OUD would have longer LOS, fewer discharges to home with services, and more patient-directed discharges and PAC discharges compared to those among patients without OUD. We also hypothesized that the charges related to inpatient hospitalization would be greater for patients with OUD, primarily driven by increased LOS.

## Methods

This study is reported as per the Strengthening the Reporting of Observational Studies in Epidemiology (STROBE) guideline (S1 STROBE Checklist). A prospective analysis plan was developed in August 2018, with analysis conducted from August to December 2018. The supplementary propensity score analysis was done in January 2019 to confirm the primary results. Additional analyses in response to peer review in March 2020 included adding the hospital as a random intercept, which did not change the estimates, and assessing changes in the logistic regression models when adding and removing covariates, which showed that controlling for age and payor primarily account for changes in our estimates (S1 Table).

### Data

We used data from the 2016 National Inpatient Sample (NIS), the largest all-payer inpatient database in the United States (US). The NIS, sponsored by the Agency for Healthcare Research and Quality, was developed under the Healthcare Cost and Utilization Project (HCUP) to include data from 20% of all hospitalizations from participating hospitals nationwide. The database contains information on approximately 8 million hospitalizations each year, with sample weights provided by HCUP allowing for estimates to be representative of the 35 million hospitalizations in the US in 2016 [20].

We included all hospitalizations for serious infections from January 1 to December 31, 2016, for adult patients 18 years or older. Serious infections included endocarditis, epidural abscess, septic arthritis, and osteomyelitis, identified using the International Classification of Diseases–10th Revision (ICD-10) diagnosis codes in the primary diagnosis field (S2 Table). We identified ICD-10 codes from prior studies of serious infections and OUD, utilizing correlates of ICD-9 codes where necessary [1,2]. We limited the codes for infections to the primary diagnosis field to improve specificity in identifying relevant hospitalizations.

### Study variables

The main exposure of interest was OUD, which was defined as hospitalizations in which a non-primary diagnosis field contained an ICD-10 code for an opioid-related disorder (S2 Table). The key outcome of interest was hospital LOS until discharge, reported in days. Secondary outcomes were discharge disposition, total hospital charges, and hospital charges per day (calculated by dividing total hospital charges by LOS).

Because we were interested in differences in healthcare utilization, independent of differences in baseline characteristics, we controlled for several covariates in our models. Patient characteristics included age, sex, race/ethnicity, primary payer, quartile of median household income based on the patient’s zip code of residence, Elixhauser Comorbidity Index (excluding drug use to avoid adjusting for our exposure of interest), and the type of serious infection. Hospital characteristics included size (based on number of beds), type of hospital (rural, urban non-teaching, or urban teaching), and US region (northeast, midwest, south, or west). Hospitalization characteristics included whether the admission was elective, whether it was on a weekend, and the number of major operating room procedures performed during the hospital stay.

### Statistical analysis

In bivariable analyses, characteristics and outcomes of hospitalizations for serious infections were compared between patients with and without OUD. Differences were examined using *t* tests for continuous variables and Rao–Scott chi-squared tests for categorical variables.

For the primary outcome of interest, LOS, we performed a competing risks survival analysis to compare time to hospital discharge between hospitalizations with and without OUD, treating death, patient-directed discharge, and transfer to another acute care hospital as competing risks. We modeled the cumulative incidence function for each cohort, using Gray’s test to assess for differences, and performed a multivariable competing risks regression analysis using the Fine–Gray subdistribution hazard function to estimate adjusted hazard ratios (aHRs) for the probability of discharge at any given time [21,22]. Additional statistical information and model specifications are detailed in S1 Text.

We fitted multivariable logistic regression models to estimate adjusted odds ratios (aORs) for each discharge disposition (home, home without health services, home with health services, PAC facility, patient-directed discharge, transfer to an acute care hospital, and death) versus the others (e.g., home versus not home). Finally, we calculated differences in hospital charges using multivariable linear regression.

All multivariable models were adjusted for the aforementioned patient, hospital, and hospitalization characteristics. The Fine–Gray model for competing risks was adjusted using the NIS survey weights; for all other models, we utilized survey analysis procedures to account for NIS survey weights, stratification, and clustering, to adjust for the complex sampling design and produce estimates representative of all US hospitalizations in 2016.

Analyses were performed using SAS 9.4 (SAS Institute). We report 95% confidence intervals for aHRs and aORs, using *p <* 0.05 to indicate statistical significance for all comparisons. The Beth Israel Deaconess Medical Center Institutional Review Board deemed this study as not human subjects research.

### Sensitivity analyses

We assessed the robustness of our findings by applying propensity score matching using a greedy match algorithm to balance covariates between our 2 cohorts, which we confirmed using standardized mean differences, and recalculated hazard and odds ratios between our matched groups.

To examine the degree to which differences are driven by decisions around home IV antibiotic administration, we performed an analysis in which we compared our outcomes of interest between hospitalizations with and without OUD for conditions not usually requiring prolonged IV access (pneumonia, acute congestive heart failure, acute cholecystitis).

To explore whether disparities in receipt of surgery may drive any observed differences in LOS, we ran stratified competing risks analyses in hospitalizations with and without any major operating room procedures during admission.

We investigated the sensitivity of our cohort definitions by examining an analytic cohort of patients hospitalized with a serious infection in any diagnosis code position. We also performed a survival analysis excluding patients who were transferred in from another hospital to examine whether left truncation might affect our results. Lastly, we examined models that adjusted for homelessness, identified using ICD-10 codes in any of the secondary diagnosis code positions (S2 Table), as well as severity of illness, as defined using All Patient Refined Diagnosis Related Groups by HCUP.

## Results

### Characteristics of hospitalizations for serious infection with and without OUD

We identified 95,470 hospitalizations with serious infection as a primary diagnosis, of which 7,635 (8.0%) had a secondary diagnosis code for OUD (OUD group), and 87,835 (92.0%) did not (non-OUD group) (Table 1). Endocarditis was the most frequent infection in the OUD group (38.4%), while osteomyelitis was most common in the non-OUD group (55.2%).

**Table 1:** Baseline patient, hospitalization, and hospital characteristics of US hospitalizations for serious infections in patients with and without opioid use disorder in 2016.

Characteristic	Opioid use disorder (*N =* 7,635)	No opioid use disorder (*N =* 87,835)	Total (*N =* 95,470)	*p-*Value
**Patient characteristics**				
Infection type, number (%)				
Infective endocarditis	2,935 (38.44)	10,195 (11.61)	13,130 (13.75)	<0.001
Epidural abscess	950 (12.44)	6,995 (7.96)	7,945 (8.32)	<0.001
Septic arthritis	1,690 (22.13)	22,135 (25.20)	23,825 (24.96)	<0.001
Osteomyelitis	2,060 (26.98)	48,510 (55.23)	50,570 (52.97)	<0.001
Age in years, mean (SE)	41.2 (0.39)	59.3 (0.15)	49.0 (0.19)	<0.001
Female, number (%)	3,355 (43.97)	30,420 (34.65)	33,775 (35.39)	<0.001
Primary payer, number (%)				
Medicare	1,230 (16.11)	42,655 (48.63)	43,885 (46.03)	<0.001
Medicaid	4,485 (58.74)	15,995 (18.24)	20,480 (21.48)	<0.001
Private	860 (11.26)	20,515 (23.39)	21,375 (22.42)	<0.001
Self-pay (uninsured)	790 (10.35)	4,810 (5.48)	5,600 (5.87)	<0.001
No charge	110 (1.44)	500 (0.57)	610 (0.64)	<0.001
Other	160 (2.10)	3,230 (3.68)	3,390 (3.56)	<0.001
Race/ethnicity, number (%)				
White	5,470 (74.02)	58,300 (69.35)	63,770 (69.73)	<0.001
Black	830 (11.23)	12,445 (14.80)	13,275 (14.52)	0.01
Hispanic	825 (11.16)	9,265 (11.02)	10,090 (11.03)	0.78
Asian or Pacific Islander	25 (0.34)	1,380 (1.64)	1,405 (1.54)	<0.001
Native American	110 (1.49)	870 (1.03)	980 (1.07)	0.13
Other	130 (1.76)	1,805 (2.15)	1,935 (2.12)	0.37
Median household income quartile, number (%)				
Quartile 1	2,780 (37.57)	28,590 (33.29)	31,370 (33.63)	0.004
Quartile 2	1,980 (26.76)	22,480 (26.17)	24,460 (26.22)	0.78
Quartile 3	1,595 (21.55)	19,360 (22.54)	20,955 (22.46)	0.31
Quartile 4	1,045 (14.12)	15,455 (17.99)	16,500 (17.69)	<0.001
Number of Elixhauser Comorbidity Index conditions, mean (SE)	2.65 (0.05)	3.34 (0.02)	3.29 (0.02)	<0.001
**Hospitalization characteristics**				
Number of major operating room procedures, mean (SE)	0.74 (0.04)	1.11 (0.01)	1.08 (0.01)	<0.001
Weekend admission, number (%)	1,780 (23.31)	15,645 (17.81)	17,425 (18.25)	<0.001
Elective admission, number (%)	505 (6.63)	14,525 (16.60)	15,030 (15.80)	<0.001
**Hospital characteristics**				
Size, number (%)				
Small	1,160 (15.19)	16,895 (19.23)	18,055 (18.91)	0.001
Medium	1,970 (25.80)	25,185 (28.67)	27,155 (28.44)	0.05
Large	4,505 (59.00)	45,755 (52.09)	50,260 (52.64)	<0.001
Urban/teaching status, number (%)				
Rural	360 (4.72)	8,190 (9.32)	8,550 (8.96)	<0.001
Urban, non-teaching	1,750 (22.92)	22,620 (25.75)	24,370 (25.53)	0.06
Urban, teaching	5,525 (72.36)	57,025 (64.92)	62,550 (65.52)	<0.001
Region, number (%)				
Northeast	2,130 (27.90)	15,510 (17.66)	17,640 (18.48)	<0.001
Midwest	1,305 (17.09)	19,305 (21.98)	20,610 (21.59)	0.002
South	2,465 (32.29)	35,180 (40.05)	37,645 (39.43)	<0.001
West	1,735 (22.72)	17,840 (20.31)	19,575 (20.50)	0.08

National estimates were generated using discharge weights computed for the 20% sample from the 2016 National Inpatient Sample. The Rao–Scott chi-squared test was used for categorical variables. The *t* test was used for continuous variables. Additional characteristics not included as covariates in the primary statistical model are listed in S3 Table. Medicare is a US federal health insurance program for people age 65 years or older. Medicaid is a US federal and state program for low-income people.

SE, standard error.

OUD hospitalizations involved patients who were younger (mean age 41.2 versus 59.3 years) and more likely to be female (44.0% versus 34.7%), be white (74.0% versus 69.4%), have Medicaid (58.7% versus 18.2%) or be uninsured (10.4% versus 5.5%), and be in the lowest quartile of median household income (37.6% versus 33.3%), compared to the non-OUD group. The OUD group had a mean of 2.7 comorbid conditions in the modified Elixhauser Comorbidity Index (excluding drug use) compared to 3.3 for the non-OUD group (see S3 Table for frequencies of the individual Elixhauser Comorbidity Index conditions as well as rates of hepatitis C infection and homelessness). The OUD group had an average of 0.7 major operating room procedures compared to 1.1 for the non-OUD group. Additionally, OUD hospitalizations were more likely to be at large, urban teaching hospitals in the northeast region. All differences were statistically significant.

### Differences in disposition

OUD hospitalizations were less likely to be discharged home (45.3% versus 63.1%), particularly with home health services (10.8% versus 27.8%), and more likely to have a patient-directed discharge (19.1% versus 2.6%) or be transferred to another acute care hospital (6.8% versus 4.5%, all *p <* 0.001) compared to the non-OUD group (Table 2).

**Table 2:** Dispositions of hospitalizations for serious infections with and without opioid use disorder.

Disposition	Opioid use disorder (*N =* 7,610)	No opioid use disorder (*N =* 87,739)	Total (*N =* 95,349)	Unadjusted odds ratio (95% CI)	*p-*Value	Adjusted odds ratio (95% CI)	*p-*Value
Home							
All	3,450 (45.3)	55,390 (63.1)	58,840 (61.7)	0.49 (0.43, 0.54)	<0.001	0.38 (0.33, 0.43)	<0.001
Without services	2,625 (34.5)	31,010 (35.3)	33,635 (35.3)	0.98 (0.87, 1.10)	0.69	0.74 (0.64, 0.84)	<0.001
With services	825 (10.8)	24,380 (27.8)	25,205 (26.4)	0.31 (0.26, 0.37)	<0.001	0.37 (0.30, 0.45)	<0.001
Post-acute care facility (rehabilitation center, skilled nursing facility)	2,120 (27.9)	25,130 (28.6)	27,250 (28.6)	0.96 (0.84, 1.11)	0.61	1.85 (1.57, 2.17)	<0.001
Transferred to another acute care facility	520 (6.8)	3,935 (4.5)	4,455 (4.7)	1.62 (1.30, 2.02)	<0.001	0.90 (0.70, 1.16)	0.41
Patient-directed discharge	1,450 (19.1)	2,310 (2.6)	3,760 (3.9)	8.82 (7.43, 10.48)	<0.001	3.47 (2.80, 4.29)	<0.001
Died	70 (0.9)	974 (1.1)	1044 (1.1)	0.84 (0.48, 1.46)	0.53	0.97 (0.52, 1.80)	0.92

Data are given as number (%). Services for home discharges include a home IV provider or being under the care of an organized home health service organization. Post-acute care facilities include skilled nursing facilities, intermediate care facilities, inpatient rehabilitation facilities, hospice facilities, long-term care hospitals, and psychiatric hospitals. National estimates generated using discharge weights computed for the 20% sample from the 2016 National Inpatient Sample. Adjusted odds ratios, 95% confidence intervals, and *p*-values were calculated using multivariable logistic regression models to reflect the odds of each disposition compared with all other dispositions in patients with opioid use disorder versus no opioid use disorder. Multivariable models were adjusted for age, sex, race/ethnicity, primary payer, median household income, Elixhauser Comorbidity Index, infection type, hospital size, hospital type, hospital region, elective versus non-elective admission, weekday versus weekend admission, and the number of major operating room procedures. Patient-directed discharge is coded as “against medical advice.”

Multivariable models were adjusted for age, sex, race/ethnicity, primary payer, quartile of median household income based on the patient’s zip code of residence, Elixhauser Comorbidity Index (excluding drug use to avoid adjusting for our exposure of interest), type of serious infection, hospital size (based on number of beds), type of hospital (rural, urban non-teaching, or urban teaching), hospital region (northeast, midwest, south, or west), elective versus non-elective admission, weekday versus weekend admission, and the number of major operating room procedures performed during the hospital stay. After adjusting for these covariates, OUD hospitalizations were less likely to be discharged to home with or without services (aOR 0.38; 95% CI 0.33–0.43; *p <* 0.001) and more likely to be discharged to a PAC facility (aOR 1.85; 95% CI 1.57–2.17; *p <* 0.001) or have a patient-directed discharge (aOR 3.47; 95% CI 2.80–4.29; *p <* 0.001) compared to non-OUD hospitalizations. There were no significant differences in the odds of being transferred to another acute care hospital or in-hospital mortality.

### Length of stay

**Fig 1:**
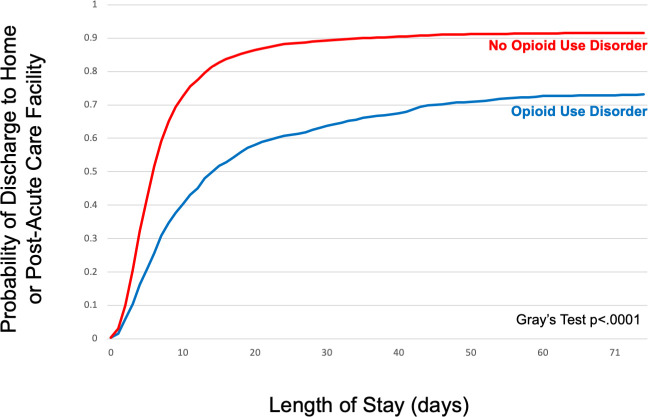
Probability of discharge to home or a post-acute care facility at any given length of stay for US hospitalizations for serious infections in patients with and without opioid use disorder in 2016. Cumulative incidence curves of length of stay to discharge estimated using a competing risks survival analysis model. The event of interest was defined as discharge to home or a post-acute care facility. Competing risks were defined as patient-directed discharge, transfer to another acute care hospital, or in-hospital death.

The mean LOS was 12.5 days for the OUD group compared to 8.1 days for the non-OUD group (difference 4.3 [95% CI 3.6–5.1]) (Table 3). When stratified by disposition location, the LOS for the OUD group was longer than for the non-OUD group for discharges to home without services (mean 15.5 versus 6.8 days; difference 8.7 [95% CI 7.2–10.2]), home with services (10.8 versus 7.6; difference 3.2 [95% CI 1.6–4.8]), and PAC facilities (13.8 versus 10.5; difference 3.3 [95% CI 2.3–4.4], all *p <* 0.001). In a survival analysis, there were significant differences in LOS (time to hospital discharge) between hospitalizations with and without OUD for all infection types (Fig 1; S1 Fig).

**Table 3:** Differences in LOS by disposition for US hospitalizations for serious infections in patients with and without opioid use disorder in 2016.

Disposition	LOS, in days, mean (SE)		Difference in LOS, in days, mean (SE)	*p-*Value
	Opioid use disorder (*N =* 7,610)	No opioid use disorder (*N =* 87,739)		
All dispositions	12.48 (0.37)	8.14 (0.09)	4.34 (0.37)	<0.001
Home				
All	14.39 (0.63)	7.15 (0.10)	7.24 (0.62)	<0.001
Without services	15.52 (0.77)	6.79 (0.13)	8.73 (0.76)	<0.001
With services	10.79 (0.84)	7.60 (0.12)	3.19 (0.84)	<0.001
Post-acute care facility	13.81 (0.54)	10.48 (0.15)	3.34 (0.54)	<0.001
Transferred to another acute care facility	7.55 (0.84)	6.32 (0.28)	1.23 (0.89)	0.17
Patient-directed discharge	7.76 (0.57)	5.40 (0.39)	2.36 (0.71)	<0.001
Died	14.14 (5.06)	17.93 (2.24)	−3.79 (5.51)	0.49

Differences in LOS are not adjusted for differences in baseline characteristics. Services for home discharges include a home IV provider or being under the care of an organized home health service organization. Post-acute care facilities include skilled nursing facilities, intermediate care facilities, inpatient rehabilitation facilities, hospice facilities, long-term care hospitals, and psychiatric hospitals. LOS represents the number of midnights crossed during a hospitalization. Patient-directed discharge is coded as “against medical advice.” *p*-Values were calculated using the Student’s *t* test.

LOS, length of stay; SE, standard error.

**Fig 2:**
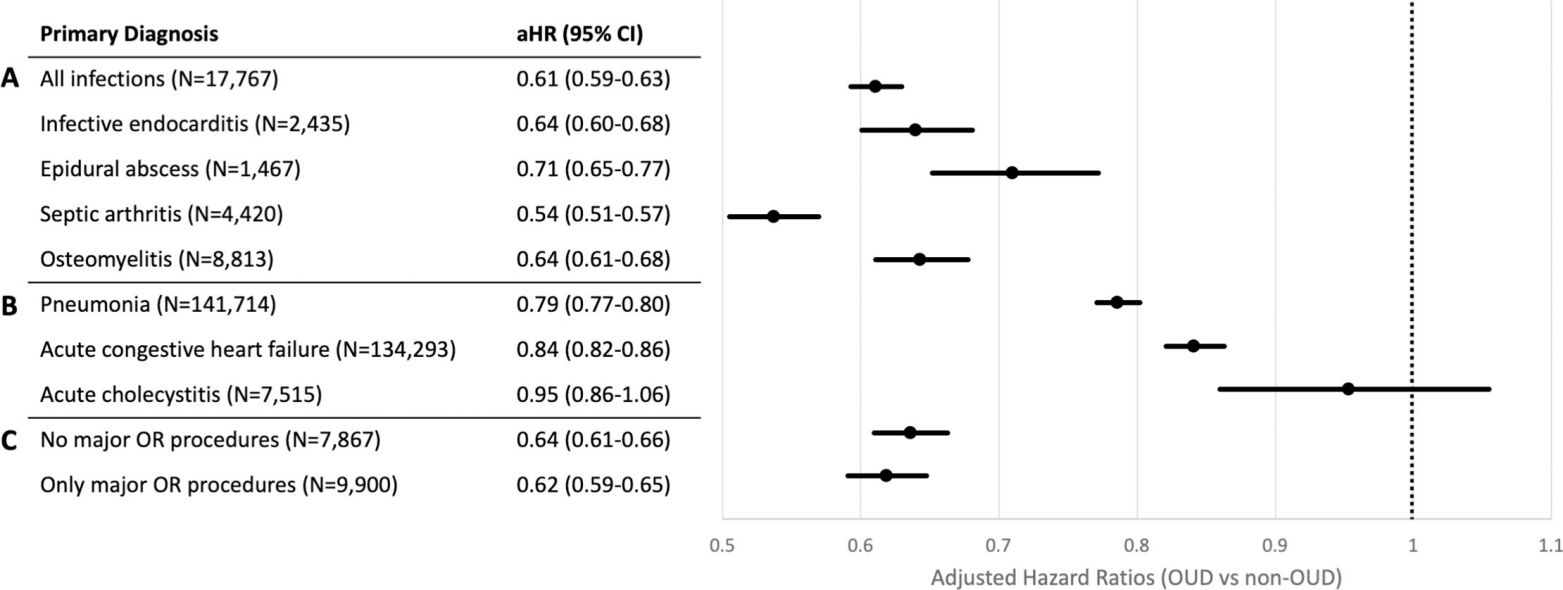
Adjusted hazard ratios of length of stay until discharge for US hospitalizations for serious infections in patients with and without opioid use disorder in 2016. Hazard ratios are from the Fine–Gray subdistribution hazard regression model, adjusted for age, sex, race/ethnicity, primary payer, median household income, Elixhauser Comorbidity Index, hospital size, hospital type, hospital region, elective versus non-elective admission, weekday versus weekend admission, and the number of major operating room procedures. The model for serious infections also controlled for infection type. The event of interest was defined as discharge to home or a post-acute care facility. Competing risks were defined as patient-directed discharge, transfer to another acute care hospital, or in-hospital death. Hazards ratios were calculated for (A) the analysis of serious infections, (B) a sensitivity analysis of conditions not usually requiring prolonged intravenous access, and (C) a sensitivity analysis stratifying the primary model by hospitalizations with and without major operating room procedures. Unadjusted hazard ratios are found in S4 Table. aHR, adjusted hazard ratio; OR, operating room; OUD, opioid use disorder.

Individuals in the OUD group had a 39% lower probability of discharge at any given LOS (aHR 0.61; 95% CI 0.59–0.63; *p <* 0.001) compared to individuals in the non-OUD group (Fig 2) after adjusting for age, sex, race/ethnicity, primary payer, median household income, Elixhauser Comorbidity Index, infection type, hospital size, hospital type, hospital region, elective versus non-elective admission, weekday versus weekend admission, and the number of major operating room procedures.

### Total and daily hospital charges

Using survey weights to calculate a nationally representative estimate, total charges for hospitalizations for serious infections in the US in 2016 were $7.15 billion, with the OUD group accounting for $739 million. The unadjusted average total charge for OUD hospitalizations was $23,948.69 higher than for non-OUD hospitalizations. However, the average daily charge was $1,016.17 less per hospital day for the OUD group compared to the non-OUD group. The largest difference in charges was seen in hospitalizations that ended in discharge home without services. For these, the OUD group had a mean total charge that was $44,498.16 greater than that of the non-OUD group, yet the average daily charge for the OUD group was $2,314.04 less per day than for the non-OUD group (Table 4).

**Table 4:** Unadjusted and adjusted total and daily hospital charges of hospitalizations for serious infections with and without opioid use disorder.

Disposition	Hospital charges in US dollars, mean (SE)				Unadjusted difference in US dollars, mean (SE)				Adjusted difference in US dollars, mean (SE)			
	Opioid use disorder (*N =* 7,610)		No opioid use disorder (*N =* 87,739)									
	Total	Per day	Total	Per day	Total charges	*p-*Value	Per day charges	*p-*Value	Total charges	*p-*Value	Per day charges	*p-*Value
All dispositions	98,207.00 (3,707.52)	8,963.06 (223.24)	74,258.00 (1,282.46)	9,979.23 (120.08)	23,948.69 (2,902.83)	<0.001	−1,016.17 (175.26)	<0.001	2,189.04 (3,231.93)	0.5	−1,637.77 (186.50)	<0.001
Home												
All	100,540.00 (5,665.75)	7,989.47 (242.22)	62,973.00 (1,239.34)	9,934.25 (138.03)	37,566.83 (4,056.03)	<0.001	−1,944.78 (202.97)	<0.001	16,387.49 (3,867.36)	<0.001	−2,093.54 (229.06)	<0.001
Without services	101,429.00 (6,748.22)	7,482.96 (260.13)	56,931.00 (1,407.04)	9,797.00 (147.55)	44,498.16 (4,930.52)	<0.001	−2,314.04 (229.50)	<0.001	15,862.31 (4,744.60)	0.001	−2,136.02 (254.36)	<0.001
With services	97,683.00 (9,435.41)	9,613.47 (548.32)	70,686.00 (1,766.37)	10,108.00 (186.38)	26,996.94 (6,460.27)	<0.001	−494.94 (425.83)	0.25	19,190.07 (6,574.57)	0.004	−919.02 (460.03)	0.05
Post-acute care facility	123,936.00 (6,537.31)	9,199.30 (363.76)	99,510.00 (2,320.32)	9,685.69 (146.20)	24,425.09 (5,756.65)	<0.001	−486.39 (317.10)	0.13	−15,318.36 (7,911.10)	0.05	−1,358.36 (349.11)	<0.001
Transferred to another acute care facility	85,273.00 (11,299.00)	12,705.00 (1089.60)	57,785.00 (3,810.10)	10,975.00 (430.70)	27,487.48 (9,915.20)	0.006	1,730.06 (776.35)	0.03	13,460.67 (12,162.26)	0.27	−184.62 (922.69)	0.84
Patient-directed discharge	55,765.00 (4,242.05)	9,113.05 (455.69)	45,234.00 (2,957.42)	10,804.00 (482.19)	10,530.91 (4,786.69)	0.03	−1,691.26 (537.23)	0.002	9,712.80 (4,720.28)	0.04	−1,976.90 (830.41)	0.02
Died	202,731.00 (62,245.00)	21,382.00 (3,791.22)	198,936.00 (20,763.00)	14,902.00 (1,202.36)	3,794.65 (64,631.48)	0.95	6,480.22 (3,558.51)	0.07	−96,882.69 (77,906.76)	0.21	431.90 (3,931.55)	0.91

Charges represent the hospital billing for the hospital stay. Charges per hospitalization day were calculated by dividing total charges by length of stay. Adjusted mean charges, standard errors, and *p*-values were calculated using multivariable linear regression models. Multivariable models were adjusted for age, sex, race/ethnicity, primary payer, median household income, Elixhauser Comorbidity Index, infection type, hospital size, hospital type, hospital region, elective versus non-elective admission, weekday versus weekend admission, and the number of major operating room procedures. Patient-directed discharge is coded as “against medical advice.”

SE, standard error.

When adjusted for covariates, there was no statistically significant difference in total hospital charges (difference $2,189.04 [95% CI −$4,145.54 to $8,523.62], *p =* 0.50), but average daily charges were significantly less for OUD hospitalizations (difference −$1,637.77 [95% CI −$2,003.47 to −$1,272.07], *p <* 0.001). Among discharges to home without services, OUD hospitalizations had higher average total charges (difference $15,862.31 [95% CI $6,558.96 to $25,165.67], *p <* 0.001) and lower average daily charges (difference −$2,136.02 [95% CI −$2,814.78 to −$1,817.25], *p <* 0.001). The covariates for the multivariable models were age, sex, race/ethnicity, primary payer, median household income, Elixhauser Comorbidity Index, infection type, hospital size, hospital type, hospital region, elective versus non-elective admission, weekday versus weekend admission, and the number of major operating room procedures.

### Sensitivity analyses

In a propensity score matched analysis, both OUD and non-OUD hospitalizations had balanced frequencies of all measured baseline covariates (standardized mean difference < 0.1; S5 Table). A competing risks regression model using the matched cohorts showed that OUD hospitalizations had a 35% lower probability of discharge at any given LOS (aHR 0.65; 95% CI 0.63–0.68; *p <* 0.001) compared to non-OUD hospitalizations—almost identical to the aHR in the main analysis (S6 Table).

Next, we evaluated hospitalizations for conditions not typically associated with a need for prolonged IV access (pneumonia, acute congestive heart failure, and acute cholecystitis) (Fig 2). The difference in the probability of discharge at any given LOS between OUD and non-OUD hospitalizations was attenuated among those hospitalized for conditions not typically associated with prolonged IV access (5%–21% difference) relative to hospitalizations for conditions associated with prolonged IV access (29%–46% difference) after adjusting for age, sex, race/ethnicity, primary payer, median household income, Elixhauser Comorbidity Index, infection type, hospital size, hospital type, hospital region, elective versus non-elective admission, weekday versus weekend admission, and the number of major operating room procedures.

Results of our primary analysis did not differ by presence or absence of any major surgical procedures (Fig 2). For hospitalizations with no procedures, OUD hospitalizations had a 36% lower probability of discharge at any given LOS compared to non-OUD hospitalizations. For hospitalizations with procedures, OUD hospitalizations had a 38% lower probability of discharge at any given LOS than non-OUD hospitalizations.

Neither expanding the definition of serious infections to include an infection in any diagnosis code position nor excluding patients who were transferred in from another hospital meaningfully changed our primary results. Additionally, adding homelessness or severity of illness as covariates did not significantly alter our findings (S1 Table).

## Discussion

In this large, nationally representative study of inpatient hospitalizations from 2016, we found patients with OUD who were hospitalized for serious infections, compared to those without OUD, had longer LOS, were less likely to be discharged home, were more likely to be discharged to a PAC facility or have a patient-directed discharge, and had similar total hospital charges despite lower daily charges. Our results were robust to multiple analytic approaches and sensitivity analyses.

To our knowledge, this is the first national study to assess differences in hospital and PAC utilization between patients with and without OUD who are hospitalized for conditions requiring prolonged IV access. Our work is consistent with prior studies showing differential patterns of healthcare utilization and longer inpatient hospital stays for patients with OUD [2,3,5,18,19]. We build on these studies by exploring a variety of serious infections that disproportionately affect this population and demonstrating nationwide disparities in care even when accounting for several potential confounders. Thus, our study provides new insights into patterns of healthcare utilization for this costly downstream complication of the ongoing opioid crisis.

Our study suggests that patients with OUD were less likely to be discharged home, especially with home health services, and more likely to be discharged to a PAC facility, despite being younger, having fewer comorbidities, and undergoing fewer surgical procedures—characteristics usually not associated with longer hospital stays and discharges to PAC facilities [23–25]. This suggests healthcare providers and hospitals are discharging patients with OUD to PAC facilities rather than home, independent of typical reasons for requiring rehabilitation or skilled nursing facilities. Inpatient providers’ concerns about discharging patients with OUD to home with an IV line may contribute to these differences [12,13]. This hypothesis is bolstered by the finding that a significantly lower proportion of patients with OUD were discharged with home health services such as visiting nursing care or home IV services. Recent evidence has demonstrated the potential effectiveness and safety of outpatient parenteral antibiotic therapy (OPAT) among people who inject drugs [8,10,26–30]. Though additional evaluations are necessary, there may be opportunities to implement more OPAT in patients with OUD. There are also promising developments that could help narrow disparities in discharge dispositions, such as residential addiction treatment facilities that could be both safe and cost-saving, inpatient addiction medicine consultations that may improve antibiotic therapy completion rates and reduce readmissions, and, in select cases, the possibility that serious infections may be safely treated with partial oral antibiotic therapy [15,31–35].

Patients with OUD had significantly longer LOS than patients without OUD. There are several potential explanations for this. First, inpatient providers may keep patients with OUD in the hospital to complete their antibiotic course owing to concerns about discharging these patients with an IV line, as described above [12]. Second, these patients may have less access to PAC facilities or home IV services, thus limiting their potential discharge options [13]. Third, patients with OUD may have a higher severity of illness. This last hypothesis is less likely since patients with OUD were younger and had fewer comorbidities, fewer procedures, fewer average daily charges, and a lower proportion of inpatient mortality. Moreover, in our sensitivity analyses, we found that the disparity in LOS for OUD compared to non-OUD hospitalizations was attenuated when focusing on conditions that do not typically require prolonged IV access such as pneumonia or acute cholecystitis. This suggests that the need for ongoing IV access is a key driver of decisions around the appropriate setting of care for these patients and contributes to the observed disparities in LOS and disposition. The attenuated but still significant differences for pneumonia and acute congestive heart failure suggest that patients with OUD have prolonged hospital stays for other reasons as well. This could be due to inpatient complications with opioid withdrawal, higher severity of illness, or lack of access to necessary discharge options. Research on recent policy efforts to improve access to PAC facilities for OUD patients may provide valuable insights into the differences in healthcare utilization [36–39].

Another important finding is the significantly higher incidence of patient-directed discharges (or “discharges against medical advice”) in the OUD group than the non-OUD group. Patient-directed discharges are a critical issue, especially due to their association with higher costs, readmission rates, and mortality [40]. Inpatient withdrawal management, substance use treatment, and social support are associated with lower likelihood of patient-directed discharge in patients with drug use [35,41,42]. These interventions are underutilized, but several successful models of inpatient withdrawal management and linkage to outpatient addiction treatment are emerging and may help to address the problem of patient-directed discharges in this patient population [43–49].

Lastly, we found that total adjusted hospital charges were similar between OUD and non-OUD hospitalizations, despite significantly lower average daily charges. The lower daily charges despite similar total charges for patients with OUD (or higher total charges for specific dispositions such as discharge home) suggest that LOS is an important driver of cost in OUD hospitalizations and reinforce the notion that patients with OUD have lower acuity and receive fewer hospital services but have comparable or more expensive hospital stays due to longer LOS. Given the disproportionate burden of these hospitalizations among patients on Medicaid and uninsured patients, there are serious and potentially preventable costs for patients, governments, insurance companies, and health systems.

Our study had some notable strengths and limitations. First, this was a cross-sectional observational analysis, so it could not demonstrate causal relationships or account for potential sources of unmeasured confounding. However, our results were robust to multiple sensitivity analyses. Additionally, the NIS allowed for the most comprehensive, high-quality, and validated national survey of inpatient hospitalizations. Second, the need to use ICD-10 codes in NIS likely led to measurement bias and underestimated cases of serious infections and OUD, as demonstrated in a recent study using the related HCUP State Inpatient Databases [50]. Though this had the disadvantage of lowering our population estimates of the various conditions, it also likely biased our results towards the null, which further highlights our significant findings. There is also the possibility of differential misclassification of potential confounders, which may have biased our results. Third, there has been a recent rise, particularly from 2017 onwards, in stimulant use, with a related rise in morbidity and mortality, which may affect decisions around hospitalizations and discharges. We did not account for stimulant use as our data focused on 2016, likely preceding this “fourth wave.” Lastly, homelessness is a major problem among patients with OUD, which could affect discharge decisions and access to PAC facilities and could lengthen hospital stays [51–53]. We sought to adjust for this by running a sensitivity analysis using homelessness as a covariate as described above, which did not significantly affect our results.

Given our findings of longer LOS for patients with OUD, differential disposition, and associated hospital charges, an important next step would be a mixed-methods study to understand the reasons for prolonged hospital stays for these patients. Additionally, more research is needed to assess the potential impact of OPAT, partial oral antibiotic therapy, and residential addiction treatment facilities in reducing hospital stays, lowering costs, and maintaining or improving outcomes of serious infections. Meanwhile, all hospitals caring for patients with substance use disorder should implement inpatient addiction medicine consultations, withdrawal management, substance use treatment, and linkage to outpatient addiction treatment, which may improve antibiotic therapy completion rates and reduce patient-directed discharges and readmissions. Additionally, recent policy efforts to improve access to PAC facilities for OUD patients should be evaluated. Policymakers can seek to lift barriers for OUD patients in accessing PAC facilities while providing more funding for PAC facilities to support and care for patients with OUD. Beyond that, hospitals and policymakers should work together to expand the number of discharge options for patients requiring prolonged IV therapy, including housing for patients with housing instability. In this way, the US health system can provide equitable care to patients with serious infections so that they can avoid costly and lengthy hospitalizations that increase their risk of nosocomial infections, deconditioning, and inpatient complications.

In conclusion, we found significant disparities between patients with and without OUD who were hospitalized for serious infections. Patients with OUD stayed in the hospital longer than those without OUD, and they had a higher odds of going to a PAC facility or choosing a patient-directed discharge, despite there being no known evidence that a shorter hospital stay and outpatient IV antibiotic therapy would lead to worse outcomes. Further studies elucidating the source of these disparities, and health systems and policy interventions to address them, may reduce hospital stays, lower costs, and improve the equity of care for patients affected by the opioid crisis.

## Supporting information

S1 STROBE Checklist Checklist of items that should be included in reports of observational studies. (DOCX)

S1 Fig Cumulative incidence curves of length of stay to discharge by infection type. Cumulative incidence curves of length of stay to discharge estimated using a competing risks survival analysis model. The event of interest was defined as discharge to home or a post-acute care facility. Competing risks were defined as discharge against medical advice, transfer to another acute care hospital, or in-hospital death. Gray’s test was used to assess for statistically significant differences in cumulative incidence between the 2 cohorts. Shaded regions indicate 95% confidence bounds.
(DOCX)

S1 Table Additional models with hospital as a random intercept, model covariates, severity of illness, and homelessness. Covariates for all adjusted models (unless otherwise specified) were age, sex, race/ethnicity, primary payer, quartile of median household income based on the patient’s zip code of residence, Elixhauser Comorbidity Index (excluding drug use to avoid adjusting for our exposure of interest), type of serious infection, hospital size (based on number of beds), type of hospital (rural, urban non-teaching, or urban teaching), hospital region (northeast, midwest, south, west), elective versus non-elective admission, weekday versus weekend admission, and the number of major operating room procedures performed during the hospital stay. SE, standard error.
(DOCX)

S2 Table ICD-10 codes for diagnoses. ICD-10 codes from the Centers for Disease Control and Prevention ICD-10-CM browser tool, available at https://icd10cmtool.cdc.gov/. The ICD-10 codes for serious infection (endocarditis, epidural abscess, septic arthritis, and osteomyelitis) were identified in the primary diagnosis code position (first out of 30 possible codes) for the main analysis. Opioid use disorder was defined as having a corresponding ICD-10 code as a secondary diagnosis code (any of diagnosis code positions 2 to 30). Pneumonia, acute congestive heart failure, and acute cholecystitis were identified in the primary diagnosis code position, for a sensitivity analysis. Lastly, homelessness, used as a covariate in a sensitivity analysis, was defined using secondary diagnosis codes.
(DOCX)

S3 Table Baseline characteristics of hospitalizations for serious infections with and without opioid use disorder—individual Elixhauser Comorbidity Index conditions, hepatitis C virus infection, and homelessness. National estimates were generated using discharge weights computed for the 20% sample from the 2016 National Inpatient Sample. The Rao–Scott chi-squared test was used to compare differences between the 2 cohorts.
(DOCX)

S4 Table Unadjusted hazard ratios of length of stay until discharge for US hospitalizations for serious infections in patients with and without opioid use disorder in 2016. Hazard ratios are from the Fine–Gray subdistribution hazard regression model. The event of interest was defined as discharge to home or a post-acute care facility. Competing risks were defined as patient-directed discharge, transfer to another acute care hospital, or in-hospital death.
(DOCX)

S5 Table Baseline characteristics for propensity score matched cohorts. Propensity scores for having opioid use disorder were generated using survey-weighted logistic regression, adjusting for all patient, hospitalization, and hospital characteristics listed in the table. The 2 cohorts were then matched using a greedy match algorithm to produce balanced cohorts of 6,605 weighted hospitalizations each. Standardized mean differences were then calculated across the cohorts by the baseline characteristics, with an absolute difference of less than 0.1 as a threshold for balance between the 2 cohorts.
(DOCX)

S6 Table Hazard ratio for the probability of discharge at any given length of stay and odds ratios for dispositions after propensity score matching. Propensity scores for having opioid use disorder were generated using survey-weighted logistic regression, adjusting for age, sex, race/ethnicity, primary payer, median household income, Elixhauser Comorbidity Index, infection type, hospital size, hospital type, hospital region, elective versus non-elective admission, weekday versus weekend admission, and the number of major operating room procedures. The 2 cohorts were then matched using a greedy match algorithm to produce balanced cohorts of 6,605 weighted hospitalizations each. Adjusted odds ratios, 95% confidence intervals, and *p-*values were calculated using multivariable logistic regression models to reflect the odds of each disposition compared with all other dispositions in patients with opioid use disorder versus no opioid use disorder. Hazard ratios were calculated from the Fine–Gray subdistribution hazard regression model. The event of interest was defined as discharge to home or a post-acute care facility. Competing risks were defined as discharge against medical advice, transfer to another acute care hospital, or in-hospital death.
(DOCX)

S1 Text Statistical analysis and model specification. (DOCX)

## Abbreviations

aHR: adjusted hazard ratio
aOR: adjusted odds ratio
HCUP: Healthcare Cost and Utilization Project
ICD-10: International Classification of Diseases–10th Revision
IV: intravenous
LOS: length of stay
NIS: National Inpatient Sample
OPAT: outpatient parenteral antibiotic therapy
OUD: opioid use disorder
PAC: post-acute care
