# Supplementary material for: Disparities in United States hospitalizations for serious infections in patients with and without opioid use disorder: A nationwide observational study
Source: PLoS Med. 2020 Aug 7;17(8):e1003247. doi: 10.1371/journal.pmed.1003247 (PMC7413412; doi:10.1371/journal.pmed.1003247)
Supplement: S1 Fig — Cumulative incidence curves of length of stay to discharge estimated using a competing risks survival analysis model. The event of interest was defined as discharge to home or a post-acute care facility. Competing risks were defined as discharge against medical advice, transfer to another acute care hospital, or in-hospital death. Gray’s test was used to assess for statistically significant differences in cumulative incidence between the 2 cohorts. Shaded regions indicate 95% confidence bounds. (DOCX) [file pmed.1003247.s002.docx]

**S1 Fig. Cumulative Incidence Curves of Length of Stay to Discharge by Infection Type**


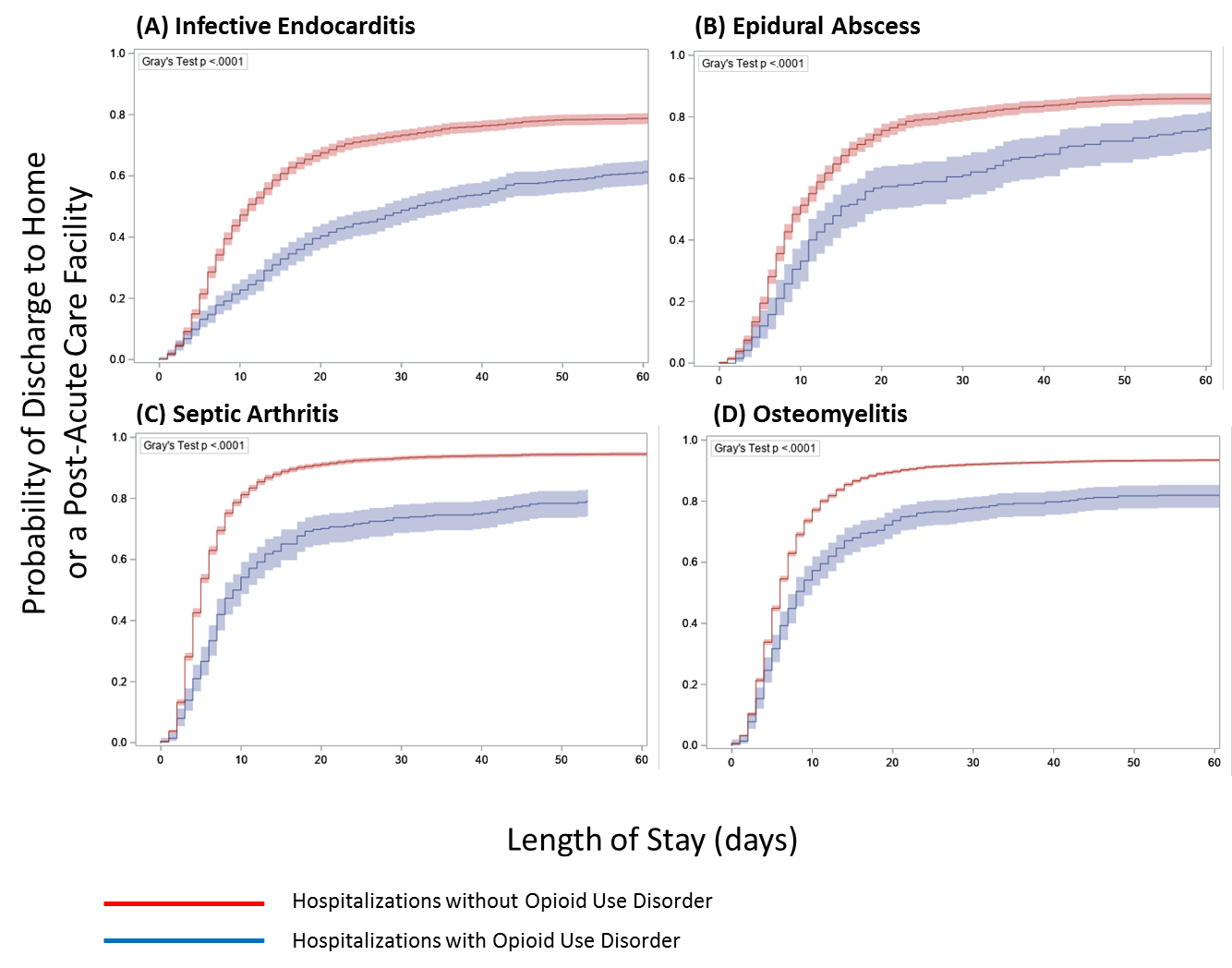


Cumulative incidence curves of length of stay to discharge estimated using a competing risks survival analysis model. The event of interest was defined as discharge to home or a post-acute care facility. Competing risks were defined as discharge against medical advice, transfer to another acute care hospital, or in-hospital death. Gray’s test was used to assess for statistically significant differences in cumulative incidence between the 2 cohorts. Shaded regions indicate 95% confidence bounds.
