# Supplementary material for: Disparities in United States hospitalizations for serious infections in patients with and without opioid use disorder: A nationwide observational study
Source: PLoS Med. 2020 Aug 7;17(8):e1003247. doi: 10.1371/journal.pmed.1003247 (PMC7413412; doi:10.1371/journal.pmed.1003247)
Supplement: S1 Table — Covariates for all adjusted models (unless otherwise specified) were age, sex, race/ethnicity, primary payer, quartile of median household income based on the patient’s zip code of residence, Elixhauser Comorbidity Index (excluding drug use to avoid adjusting for our exposure of interest), type of serious infection, hospital size (based on number of beds), type of hospital (rural, urban non-teaching, or urban teaching), hospital region (northeast, midwest, south, west), elective versus non-elective admission, weekday versus weekend admission, and the number of major operating room procedures performed during the hospital stay. SE, standard error. (DOCX) [file pmed.1003247.s003.docx]

**S1 Table. Additional Models with Hospitals as a Random Intercept, Model Covariates, Severity of Illness, and Homelessness**

**(A) Hospitals as a Random Intercept**

Hazard ratio of discharge to home or post-acute care

Independent variable: presence or absence of opioid use disorder (OUD)

|  | **Unadjusted Hazard Ratio of Discharge to Home or Post-Acute Care (95% CI)** | **P-value** | **Adjusted Hazard Ratio of Discharge to Home or Post-Acute Care (95% CI)*** | **P-value** |
| --- | --- | --- | --- | --- |
| Without hospitals as a random intercept | 0.49 (0.48, 0.50) | <0.001 | 0.61 (0.59, 0.63) | <0.001 |
| With hospitals as a random intercept | 0.49 (0.48, 0.50) | <0.001 | 0.61 (0.59, 0.63) | <0.001 |

Mean difference in total hospital charges

Independent variable: presence or absence of opioid use disorder

|  | **Unadjusted Mean Difference in Total Hospital Charges, US$ (SE)** | **P-value** | **Adjusted Mean Difference in Total Hospital Charges, US$ (SE)** | **P-value** |
| --- | --- | --- | --- | --- |
| Without hospitals as a random intercept | 23,948.69 (2902.83) | <0.001 | -2,189.04 (3231.93) | 0.50 |
| With hospitals as a random intercept | 24,921.37 (2881.05) | <0.001 | -2,905.29 (3171.54) | 0.36 |

**(B) OUD with Model Covariates for Odds Ratio of Discharge to a Post-Acute Care Facility**

Independent variable: presence or absence of opioid use disorder

|  | **Odds Ratio of Discharge to a Post-Acute Care Facility (95% CI)** | **P-value** |
| --- | --- | --- |
| OUD + no covariates | 0.96 (0.84, 1.11) | 0.61 |
| OUD + age | 2.04 (1.75, 2.36) | <0.001 |
| OUD + payor | 1.35 (1.17, 1.56) | <0.001 |
| OUD + all other covariates (individually) | *Similar odds ratio to model without covariates* | *All p>0.05* |
| OUD + all covariates | 1.85 (1.57, 2.17) | <0.001 |

**(C) Severity of Illness Added as a Covariate**

| ***Severity of Illness*** | **Unadjusted Hazard Ratio of Discharge to Home or Post-Acute Care (95% CI)** | **P-value** | **Adjusted Hazard Ratio of Discharge to Home or Post-Acute Care (95% CI)*** | **P-value** |
| --- | --- | --- | --- | --- |
| Adding APR-DRG Severity of Illness as a covariate | 0.50 (0.49, 0.52) | <0.001 | 0.63 (0.61, 0.65) | <0.001 |
| Adding APR-DRG Mortality Risk as a covariate | 0.48 (0.46, 0.49) | <0.001 | 0.61 (0.59, 0.63) | <0.001 |

**(D) Homelessness Added as a Covariate**

| ***Homelessness*** | **Unadjusted Hazard Ratio of Discharge to Home or Post-Acute Care (95% CI)** | **P-value** | **Adjusted Hazard Ratio of Discharge to Home or Post-Acute Care (95% CI)*** | **P-value** |
| --- | --- | --- | --- | --- |
| Adding homelessness as a covariate based on the ICD-10 code (S3 Appendix) | 0.50 (0.49, 0.51) | <0.001 | 0.62 (0.60, 0.64) | <0.001 |

Covariates for all adjusted models (unless otherwise specified) were age, sex, race/ethnicity, primary payer, quartile of median household income based on the patient’s zip code of residence, Elixhauser Comorbidity Index (excluding drug use to avoid adjusting for our exposure of interest), type of serious infection, hospital size (based on number of beds), type of hospital (rural, urban non-teaching, or urban teaching), hospital region (northeast, midwest, south, west), elective vs non-elective admission, weekday vs weekend admission, and the number of major operating room procedures performed during the hospital stay. SE=standard error.
