# Supplementary material for: Disparities in United States hospitalizations for serious infections in patients with and without opioid use disorder: A nationwide observational study
Source: PLoS Med. 2020 Aug 7;17(8):e1003247. doi: 10.1371/journal.pmed.1003247 (PMC7413412; doi:10.1371/journal.pmed.1003247)
Supplement: S2 Table — The ICD-10 codes for serious infection (endocarditis, epidural abscess, septic arthritis, and osteomyelitis) were identified in the primary diagnosis code position (first out of 30 possible codes) for the main analysis. Opioid use disorder was defined as having a corresponding ICD-10 code as a secondary diagnosis code (any of diagnosis code positions 2 to 30). Pneumonia, acute congestive heart failure, and acute cholecystitis were identified in the primary diagnosis code position, for a sensitivity analysis. Lastly, homelessness, used as a covariate in a sensitivity analysis, was defined using secondary diagnosis codes. (DOCX) [file pmed.1003247.s004.docx]

**S2 Table. ICD-10 Codes for Diagnoses**

| **Diagnosis** | **ICD-10 Code** | **Description** |
| --- | --- | --- |
| **Opioid use disorder** | F11.1, F11.2, F11.9 | Opioid related disorders |
| **Endocarditis** | A39.51  B37.6  I33  I33.0  I33.9  I38  I39 | Meningococcal endocarditis  Candidal endocarditis  Acute and subacute endocarditis  Acute and subacute infective endocarditis  Acute and subacute endocarditis, unspecified  Endocarditis, valve unspecified  Endocarditis and heart valve disorders in diseases classified elsewhere |
| **Epidural abscess** | G06  G06.0  G06.1  G07 | Intracranial and intraspinal abscess and granuloma  Intracranial abscess and granuloma  Intraspinal abscess and granuloma  Extradural and subdural abscess, unspecified |
| **Septic arthritis** | A02.23  A39.83  M00.0-M00.9  M01.X-M01.X79 | Salmonella arthritis  Meningococcal arthritis  Pyogenic arthritis  Direct infections of joint in infectious and parasitic diseases classified elsewhere |
| **Osteomyelitis** | A02.24  M86.00-M86.051  M86.10-M86.151  M86.20-M86.251  M86.8X-M86.8X9 | Salmonella osteomyelitis  Acute hematogenous osteomyelitis  Other acute osteomyelitis  Subacute osteomyelitis  Other osteomyelitis |
| **Pneumonia** | J09  J10  J11  J12  J13  J14  J15  J16  J17  J18 | Influenza due to certain identified influenza viruses  Influenza due to other identified influenza virus  Influenza due to unidentified influenza virus  Viral pneumonia, not elsewhere classified  Pneumonia due to Streptococcus pneumoniae  Pneumonia due to Hemophilus influenzae  Bacterial pneumonia, not elsewhere classified  Pneumonia due to other infectious organisms, not elsewhere classified  Pneumonia in disease classified elsewhere  Pneumonia, unspecified organism |
| **Acute congestive heart failure** | I50.21  I50.23  I50.31  I50.33  I50.41  I50.43 | Acute systolic (congestive) heart failure  Acute on chronic systolic (congestive) heart failure  Acute diastolic (congestive) heart failure  Acute on chronic diastolic (congestive) heart failure  Acute combined systolic (congestive) and diastolic (congestive) heart failure  Acute on chronic combined systolic (congestive) and diastolic (congestive) heart failure |
| **Acute cholecystitis** | K81.0 | Acute cholecystitis |
| **Hepatitis C virus infection** | B17.1  B18.2  B19.2 | Acute hepatitis C  Chronic viral hepatitis C  Unspecified viral hepatitis C |
| **Homelessness** | Z59.0 | Homelessness |

ICD-10 Codes from the Centers for Disease Control and Prevention ICD-10-CM Browser Tool, available at: https://icd10cmtool.cdc.gov/. The ICD-10 codes for serious infection (endocarditis, epidural abscess, septic arthritis, and osteomyelitis) were identified in the primary diagnosis code position (first out of 30 possible codes) for the main analysis. Opioid use disorder was defined as having a corresponding ICD-10 code as a secondary diagnosis code (any of diagnosis code positions 2 to 30). Pneumonia, acute congestive heart failure, and acute cholecystitis were identified in the primary diagnosis code position for a sensitivity analysis. Lastly, homelessness, used as a covariate in a sensitivity analysis, was defined using secondary diagnosis codes.
