# Supplementary material for: Disparities in United States hospitalizations for serious infections in patients with and without opioid use disorder: A nationwide observational study
Source: PLoS Med. 2020 Aug 7;17(8):e1003247. doi: 10.1371/journal.pmed.1003247 (PMC7413412; doi:10.1371/journal.pmed.1003247)
Supplement: S3 Table — The Rao–Scott chi-squared test was used to compare differences between the 2 cohorts. (DOCX) [file pmed.1003247.s005.docx]

**S3 Table. Baseline Characteristics of Hospitalizations for Serious Infections with and without Opioid Use Disorder – Individual Elixhauser Comorbidity Index Conditions, Hepatitis C Virus Infection, and Homelessness**

|  | **Opioid Use Disorder (N=7,635)** | **No Opioid Use Disorder (N=87,835)** | **Total (N=95,470)** | **P-value** |
| --- | --- | --- | --- | --- |
| **Elixhauser Comorbidity Index Conditions, no. (%)**  Congestive heart failure  Valvular disease  Pulmonary circulation disorders  Peripheral vascular disease  Hypertension  Paralysis  Other neurological disorders  Chronic pulmonary disease  Diabetes w/o complications  Diabetes w/ chronic complications  Hypothyroidism  Renal failure  Liver disease  Chronic peptic ulcer disease  HIV and AIDS  Lymphoma  Metastatic cancer  Solid tumor without metastasis  Collagen vascular diseases  Coagulation deficiency  Obesity  Weight loss  Fluid and electrolyte disorders  Blood loss anemia  Deficiency anemias  Alcohol abuse  Psychoses  Depression  **Hepatitis C virus infection, no. (%)**  **Homelessness, no. (%)** | 235 (3.08)  250 (3.27)  110 (1.44)  1,075 (14.08)  2,275 (29.80)  360 (4.72)  525 (6.88)  1,425 (18.66)  345 (4.52)  730 (9.56)  240 (3.14)  480 (6.29)  1,650 (21.61)  40 (0.52)  75 (0.98)  20 (0.26)  30 (0.39)  40 (0.52)  285 (3.73)  680 (8.91)  580 (7.60)  855 (11.20)  2,095 (27.44)  45 (0.59)  2,570 (33.66)  720 (9.43)  985 (12.90)  1,505 (19.71)  3,455 (45.25)  665 (8.71) | 10,025 (11.41)  3,680 (4.19)  485 (0.55)  15,560 (17.72)  56,660 (64.51)  6,275 (7.14)  6,550 (7.46)  14,780 (16.83)  10,210 (11.62)  30,565 (34.80)  9,780 (11.13)  19,885 (22.64)  4,465 (5.08)  580 (0.66)  295 (0.34)  620 (0.71)  900 (1.02)  1,130 (1.29)  4,880 (5.56)  4,806 (5.47)  15,360 (17.49)  6,605 (7.52)  23,010 (26.20)  830 (0.94)  26,250 (29.89)  4,305 (4.90)  3,720 (4.24)  11,460 (13.05)  4,205 (4.79)  1,580 (1.80) | 10,260 (10.75)  3930 (4.12)  595 (0.62)  16,635 (17.42)  58,935 (61.73)  6,635 (6.95)  7,075 (7.41)  16,205 (16.97)  10,555 (11.06)  31,295 (32.78)  10,020 (10.50)  20,365 (21.33)  6,115 (6.41)  620 (0.65)  370 (0.39)  640 (0.67)  930 (0.97)  1,170 (1.23)  5,165 (5.41)  5,485 (5.75)  15,940 (16.70)  7,460 (7.81)  25,105 (26.30)  875 (0.92)  28,820 (30.19)  5,025 (5.26)  4,704 (4.93)  12,965 (13.58)  7,660 (8.02)  2,245 (2.35) | <0.001  0.09  <0.001  <0.001  <0.001  <0.001  0.41  0.07  <0.001  <0.001  <0.001  <0.001  <0.001  0.53  <0.001  0.04  0.02  0.01  0.003  <0.001  <0.001  <0.001  0.28  0.16  0.003  <0.001  <0.001  <0.001  <0.001  <0.001 |

National estimates were generated using discharge weights computed for the 20% sample from the 2016 National Inpatient Sample. The Rao-Scott chi-square test was used to compare differences between the 2 cohorts.
