# Supplementary material for: Disparities in United States hospitalizations for serious infections in patients with and without opioid use disorder: A nationwide observational study
Source: PLoS Med. 2020 Aug 7;17(8):e1003247. doi: 10.1371/journal.pmed.1003247 (PMC7413412; doi:10.1371/journal.pmed.1003247)
Supplement: S4 Table — Hazard ratios are from the Fine–Gray subdistribution hazard regression model. The event of interest was defined as discharge to home or a post-acute care facility. Competing risks were defined as patient-directed discharge, transfer to another acute care hospital, or in-hospital death. (DOCX) [file pmed.1003247.s006.docx]

**S4 Table. Unadjusted Hazard Ratios of Length of Stay until Discharge for U.S. Hospitalizations for Serious Infections in Patients with and without Opioid Use Disorder in 2016**

|  | **Unadjusted Hazard Ratio (95% CI)** | **P-value** |
| --- | --- | --- |
| **(A) All serious infections**  Infective endocarditis  Epidural abscess  Septic arthritis  Osteomyelitis | 0.49 (0.48, 0.50)  0.56 (0.53, 0.59)  0.67 (0.63, 0.72)  0.51 (0.49, 0.54)  0.63 (0.60, 0.66) | <0.001  <0.001  <0.001  <0.001  <0.001 |
| **(B)**  Pneumonia  Acute congestive heart failure  Acute cholecystitis | 0.82 (0.81, 0.84)  0.82 (0.80, 0.84)  1.04 (0.95, 1.13) | <0.001  <0.001  0.44 |
| **(C)**  No major operating room procedures  Only major operating room procedures | 0.50 (0.49, 0.52)  0.54 (0.52, 0.56) | <0.001  <0.001 |

Hazard ratios are from the Fine-Gray subdistribution hazard regression model. The event of interest was defined as discharge to home or a post-acute care facility. Competing risks were defined as patient-directed discharge, transfer to another acute care hospital, or in-hospital death.
