# Supplementary material for: Disparities in United States hospitalizations for serious infections in patients with and without opioid use disorder: A nationwide observational study
Source: PLoS Med. 2020 Aug 7;17(8):e1003247. doi: 10.1371/journal.pmed.1003247 (PMC7413412; doi:10.1371/journal.pmed.1003247)
Supplement: S5 Table — Propensity scores for having opioid use disorder were generated using survey-weighted logistic regression, adjusting for all patient, hospitalization, and hospital characteristics listed in the table. The 2 cohorts were then matched using a greedy match algorithm to produce balanced cohorts of 6,605 weighted hospitalizations each. Standardized mean differences were then calculated across the cohorts by the baseline characteristics, with an absolute difference of less than 0.1 as a threshold for balance between the 2 cohorts. (DOCX) [file pmed.1003247.s007.docx]

**S5 Table. Baseline Characteristics for Propensity Score Matched Cohorts.**

|  | **Opioid Use Disorder (N=6,605)** | **No Opioid Use Disorder (N=6,605)** | **Absolute standardized mean difference** |
| --- | --- | --- | --- |
| **PATIENT-LEVEL CHARACTERISTICS** | | | |
| **Infection type, no. (%)**  Infective endocarditis  Epidural abscess  Septic arthritis  Osteomyelitis | 2,220 (33.61)  880 (13.32)  1,600 (24.22)  1,905 (28.84) | 2,255 (34.14)  805 (12.19)  1,705 (25.81)  1,840 (27.86) | 0.05 |
| **Age in yrs, mean (SD)** | 42.02 (30.06) | 42.19 (46.90) | 0.004 |
| **Female, no. (%)** | 2,760 (41.79) | 2,710 (41.03) | 0.02 |
| **Primary payer, no. (%)**  Medicare  Medicaid  Private  Self-pay (uninsured)  No charge  Other | 1,135 (17.18)  3,715 (56.25)  815 (12.34)  710 (10.75)  95 (1.44)  135 (2.04) | 1,215 (18.40)  3,690 (55.87)  820 (12.41)  710 (10.75)  65 (0.98)  105 (1.59) | 0.07 |
| **Race, no. (%)**  White  Black  Hispanic  Asian or Pacific Islander  Native American  Other | 4,805 (72.75)  790 (11.96)  775 (11.73)  25 (0.38)  105 (1.59)  105 (1.59) | 4,790 (72.52)  820 (12.41)  760 (11.51)  15 (0.23)  115 (1.74)  105 (1.59) | <0.001 |
| **Median household income, no. (%)**  Quartile 1  Quartile 2  Quartile 3  Quartile 4 | 2,535 (38.38)  1,770 (26.80)  1,400 (21.20)  900 (13.63) | 2,635 (39.89)  1,785 (27.02)  1,325 (20.06)  860 (13.02) | 0.05 |
| **No. of Elixhauser Comorbidity Index conditions, mean (SD)** | 2.66 (4.07) | 2.69 (4.76) | 0.005 |
| **HOSPITALIZATION-LEVEL CHARACTERISTICS** | | | |
| **Number of major operating room procedures, mean (SD)** | 0.77 (3.20) | 0.77 (2.68) | 0.002 |
| **Weekend admission, no. (%)** | 1,490 (22.56) | 1,585 (24.00) | 0.03 |
| **Elective admission, no. (%)** | 440 (6.66) | 420 (6.36) | 0.01 |
| **HOSPITAL-LEVEL CHARACTERISTICS** | | | |
| **Size, no. (%)**  Small  Medium  Large | 980 (14.84)  1,740 (26.34)  3,885 (58.82) | 1,010 (15.29)  1,760 (26.65)  3,835 (58.06) | 0.02 |
| **Urban/teaching status, no. (%)**  Rural  Urban, non-teaching  Urban, teaching | 295 (4.47)  1,550 (23.47)  4,760 (72.07) | 325 (4.92)  1,470 (22.26)  4,810 (72.82) | 0.05 |
| **Region, no. (%)**  Northeast  Midwest  South  West | 1,735 (26.27)  1,145 (17.34)  2,225 (33.69)  1,500 (22.71) | 1,805 (27.33)  1,045 (15.82)  2,220 (33.61)  1,535 (23.24) | 0.03 |

Propensity scores for having opioid use disorder were generated using survey-weighted logistic regression, adjusting for all patient, hospitalization, and hospital characteristics listed in the table. The 2 cohorts were then matched using a greedy match algorithm to produce balanced cohorts of 6,605 weighted hospitalizations each. Standardized mean differences were then calculated across the cohorts by the baseline characteristics, with an absolute difference of less than 0.1 as a threshold for balance between the 2 cohorts.
