# Supplementary material for: Disparities in United States hospitalizations for serious infections in patients with and without opioid use disorder: A nationwide observational study
Source: PLoS Med. 2020 Aug 7;17(8):e1003247. doi: 10.1371/journal.pmed.1003247 (PMC7413412; doi:10.1371/journal.pmed.1003247)
Supplement: S6 Table — Propensity scores for having opioid use disorder were generated using survey-weighted logistic regression, adjusting for age, sex, race/ethnicity, primary payer, median household income, Elixhauser Comorbidity Index, infection type, hospital size, hospital type, hospital region, elective versus non-elective admission, weekday versus weekend admission, and the number of major operating room procedures. The 2 cohorts were then matched using a greedy match algorithm to produce balanced cohorts of 6,605 weighted hospitalizations each. Adjusted odds ratios, 95% confidence intervals, and p-values were calculated using multivariable logistic regression models to reflect the odds of each disposition compared with all other dispositions in patients with opioid use disorder versus no opioid use disorder. Hazard ratios were calculated from the Fine–Gray subdistribution hazard regression model. The event of interest was defined as discharge to home or a post-acute care facility. Competing risks were defined as discharge against medical advice, transfer to another acute care hospital, or in-hospital death. (DOCX) [file pmed.1003247.s008.docx]

**S6 Table. Hazard Ratio for the Probability of Discharge at Any Given Length of Stay and Odds Ratios for Dispositions after Propensity Score Matching**

|  | **Hazard Ratio (95% CI)** | **P-value** |
| --- | --- | --- |
| **All serious infections** | 0.65 (0.63, 0.68) | <0.001 |
|  | **Odds Ratio (95% CI)** | **P-value** |
| **Home, no. (%)**  **All**  **Without services**  **With services** | 0.61 (0.52, 0.72)  1.00 (0.85, 1.18)  0.42 (0.33, 0.52) | <0.001  0.98  <0.001 |
| **Post-acute care facility, no. (%) (rehabilitation center, skilled nursing facility)** | 1.19 (0.98, 1.43) | 0.08 |
| **Transferred to another acute care facility, no. (%)** | 0.88 (0.65, 1.19) | 0.40 |
| **Against medical advice, no. (%)** | 3.27 (2.46, 4.34) | <0.001 |
| **Died, no. (%)** | 0.29 (0.15, 0.59) | <0.001 |

Propensity scores for having opioid use disorder were generated using survey-weighted logistic regression, adjusting for age, sex, race/ethnicity, primary payer, median household income, Elixhauser Comorbidity Index, infection type, hospital size, hospital type, hospital region, elective vs non-elective admission, weekday vs weekend admission, and the number of major operating room procedures. The 2 cohorts were then matched using a greedy match algorithm to produce balanced cohorts of 6,605 weighted hospitalizations each. Adjusted odds ratios, 95% confidence intervals, and p-values were calculated using multivariable logistic regression models to reflect the odds of each disposition compared with all other dispositions in patients with opioid use disorder vs no opioid use disorder. Hazard ratios were calculated from the Fine-Gray subdistribution hazard regression model. The event of interest was defined as discharge to home or a post-acute care facility. Competing risks were defined as discharge against medical advice, transfer to another acute care hospital, or in-hospital death.
