# Supplementary material for: Disparities in United States hospitalizations for serious infections in patients with and without opioid use disorder: A nationwide observational study
Source: PLoS Med. 2020 Aug 7;17(8):e1003247. doi: 10.1371/journal.pmed.1003247 (PMC7413412; doi:10.1371/journal.pmed.1003247)
Supplement: S1 Text — (DOCX) [file pmed.1003247.s009.docx]

**S1 Text.** Statistical Analysis and Model Specification

Effects were estimated using the general model specification below:

*Y_i_* = *β_0_ +β_1_***Opioid Use_i_ +* *β_2_***Infection Type_i_ + β_3_***Female_i_ + β_4_***Age_i_ + β_5_***Race_i_ + β_6_***Primary Payor_i_ + β_7_***Median Household Income by Patient’s Zip Code_i_ + β_8_***Number of Major Operating Room Procedures_i_ + β_9_***Hospital Bed Size_i_ + β_10_***Hospital Urban/Rural and Teaching Status_i_ + β_11_***Hospital Region_i_ + β_12_***Weekend Admission_i_ + β_13_***Elective Admission_i_ + β_14_***Number of Elixhauser Comorbidities_i_* + *ε_i_*

For the primary competing risks survival analysis model, 17,767 observations were used in the model with 16,054 experiencing the event of interest (discharge to home or post-acute care) and 1,702 experiencing a competing event (discharge against medical advice, transfer to another acute care facility, or death) and 11 censored (destination unknown). The outcome of interest was length of stay (time to discharge) while the primary predictor of interest was opioid use status. The competing risks model was weighted using survey weights from the National Inpatient Sample. Both Harrell’s and Uno’s concordance statistics for the model was 0.66. Coefficients and standard errors were exponentiated to derive adjusted hazard ratios and 95% confidence intervals as presented in Figure 2.

For the secondary multivariable logistic regression model to determine the odds of discharge dispositions, 18,855 observations were used with c-statistics ranging from 0.62 to 0.85, depending on disposition. Multiple regression models were built using discharge disposition (home with and/or without services, post-acute care facility, against medical advice, interhospital transfer, and death) as the outcome of interest with opioid use status as the primary predictor of interest. Coefficients and standard errors were exponentiated to estimate adjusted odds ratios and 95% confidence intervals, shown in Table 3.

For the secondary multivariable linear regression model to estimate differences in hospital charges, 17,465 observations were utilized. The R-squared statistic for the model including all dispositions was 0.25. Coefficients and standard errors from the linear regression models are listed in Table 4.

For the propensity score matched analysis, propensity scores for having opioid use disorder were generated using survey-weighted logistic regression, adjusting for all patient, hospitalization, and hospital-level characteristics from Table 1. The two cohorts were then matched using a greedy match algorithm to produce balanced cohorts of 6,605 weighted hospitalizations in each cohort.
